# Towards a new set of classification criteria for PFAPA syndrome

**DOI:** 10.1186/s12969-018-0277-2

**Published:** 2018-09-21

**Authors:** Federica Vanoni, Roberta Caorsi, Sandra Aeby, Marie Cochard, Jordi Antón, Stefan Berg, Riva Brik, Pavla Dolezalova, Isabelle Koné-Paut, Benedicte Neven, Seza Ozen, Pascal Pillet, Silvia Stojanov, Carine Wouters, Marco Gattorno, Michaël Hofer

**Affiliations:** 1 Istituto Pediatrico della Svizzera Italiana, Ospedale San Giovanni, 6500 Bellinzona, Switzerland; 2 Unité Romande d’Immuno-rhumatologie Pédiatrique, CHUV, University of Lausanne, Lausanne and HUG, Geneva, Switzerland; 3 Istituto Giannina Gaslini, Clinica Pediatrica e Reumatologia, PRINTO, Genoa, Italy; 4 Pediatric Rheumatology, Hospital Sant Joan de Déu. Universitat de Barcelona, Esplugues de Llobregat, Barcelona, Spain; 5 Department of Pediatrics, The Queen Silvia Children’s Hospital, Goteborg, Sweden; 6 Department of Pediatrics and Pediatric Rheumatology Service, Ruth Rappaport Children’s Hospital, Rambam Medical Center, Haifa, Israel; 7 Department of Paediatrics and Adolescent Medicine, Paediatric Rheumatology Unit, General University Hospital and 1st Faculty of Medicine, Charles University in Prague, Praha, Czech Republic; 8 Rhumatologie pédiatrique, CHU Le Kremlin Bicêtre APHP, University of Paris Sud –CEREMAIA, Paris, France; 9 Unité d’Immunologie-Hématologie et Rhumatologie Pédiatriques CHU Paris - Hôpital Necker-Enfants Malades, Paris, France; 10 Department of Pediatric Rheumatology, Hacettepe University, Ankara, Turkey; 11 Service de pédiatrie médicale CHRU de Bordeaux, Bordeaux, France; 12 Department of Infectious Diseases and Immunology, Children’s Hospital, University of Munich, Munich, Germany; 13 Department of Microbiology and Immunology, Laboratory Paediatric Immunology, UZ Leuven Hospital, Leuven, Belgium

**Keywords:** Autoinflammatory diseases, PFAPA

## Abstract

**Background:**

Diagnosis of Periodic Fever, Aphthous stomatitis, Pharyngitis and Cervical Adenitis (PFAPA) syndrome is currently based on the modified Marshall’s criteria, but no validated evidence based classification criteria for PFAPA has been established so far.

**Methods:**

A multistep process, based on the Delphi and Nominal Group Technique was conducted. After 2 rounds of e-mail Delphi survey involving 21 experts in autoinflammation we obtained a list of variables that were discussed in an International Consensus Conference. Variables reaching the 80% of consensus between participants were included in the new classification criteria.

In the second phase the new classification criteria and the modified Marshall’s criteria were applied on a cohort of 80 pediatric PFAPA patients to compare their performance.

**Results:**

The Delphi Survey was sent to 22 participants, 21 accepted to participate. Thirty variables were obtained from the survey and have been discussed at the Consensus Conference. Through the Nominal Group Technique we obtained a new set of classification criteria. These criteria were more restrictive in respect to the modified Marshall’s criteria when applied on our cohort of patients.

**Conclusion:**

Our work led us to identify a new set of classification criteria for PFAPA syndrome, but they resulted to be too restrictive to be applied in daily clinical practice for the diagnosis of PFAPA.

## Background

Periodic Fever, Aphthous stomatitis, Pharyngitis and Cervical Adenitis (PFAPA) syndrome is characterized by regularly recurrent fever flares of early onset, accompanied by pharyngitis, cervical lymphadenopathy and oral aphthous ulcers [1]. The diagnosis is currently based on the modified Marshall’s criteria proposed in 1999 [2], but the power of these criteria remains limited, and they show a good sensitivity but a lack in specificity [3]. PFAPA is not a well-defined disease and shows a clinical overlap with the inherited periodic fevers, such as Familial Mediterranean Fever, Tumor necrosis factor receptor-associated periodic syndrome and Mevalonate kinase deficiency, for which a causative gene is well established. These diseases often require a more intensive therapeutic approach and have a more severe course and long-term prognosis. Therefore among those showing a PFAPA phenotype, it would be important to identify patients at risk of carrying mutation in genes associated with inherited periodic fever to avoid unnecessary genetic testing [3]. Moreover, PFAPA shows a clinical overlap with other infectious diseases, and more specific criteria could help avoid prescriptions of unnecessary antibiotic therapies.

In 2008 we started an international collaborative effort, aimed at developing new classification criteria based on expert consensus and analysis of real patient data, to identify a new set of classification criteria for PFAPA syndrome with higher specificity. This project was conducted through several steps: 1) a preliminary Delphi survey among international pediatricians and rheumatologists; 2) a consensus conference of physicians with specific expertise in PFAPA to obtain a provisional new set of classification criteria; 3) a preliminary evaluation of the new set of criteria on a cohort of real PFAPA patients.

## Methods

At first we proceeded to the creation of the classification criteria by using the Delphi and Nominal Group Technique [4]. We performed 2 rounds of e-mail Delphi survey involving 22 experts in autoinflammation to propose criteria for the diagnosis of PFAPA: first the experts listed the most relevant variables in their opinion for the diagnosis of PFAPA, and the proposed variables were sorted in 8 categories; secondly, the experts had to validate categories and individual items by groups of symptoms ranking the criteria. Variables coming from this survey were discussed in an International Conference that was held in Morges, Switzerland, in November 2008, with 11 experts. By using the Nominal Group technique, each variable included in the list was discussed and included in the classification criteria if a consensus of 80% was reached.

In the second phase we aimed to evaluate the new set of criteria on a group of patients with a diagnosis of PFAPA given at a specialized clinic for pediatric rheumatology. Thus, we applied the new set of classification criteria and the modified Marshall’s criteria on a cohort of 80 pediatric PFAPA patients from Lausanne (Switzerland) and Genoa (Italy) pediatric rheumatology units to compare their performance. For this study we selected all the patients with a diagnosis of PFAPA validated by an experienced pediatric rheumatologist (MH and MG), with complete information on variables included in both sets of criteria.

## Results

The Delphi Survey was sent to 22 participants, 21 accepted to participate, one didn’t respond.

Seventy-two different variables were obtained from the first survey, and could be sorted in 8 different categories. After the second Delphi, 6 categories reached consensus, and 2 individual variables; the other categories and variables have been discussed at the Consensus Conference (Table 1).

**Table 1:** Categories and variables reaching consensus during both Delphi

Categories/variables	Consensus (80%)
1. Recurrent fever attacks	Delphi 1
Duration of attacks	no
Regularity of fever attacks	no
2. Constitutional symptoms	Delphi 1
Actual criterion^a^	no
New criterion^b^	Delphi 2
3. Exclusion	Delphi 1
Exclusion of autoinflammatory diseases	Delphi 2
Exclusion of infections and related conditions	Delphi 2
4. Age limitation for onset	no
5. Well-being between episodes	Delphi 2
6. Growth and development	Delphi 2
7. Response to steroid treatment	Delphi 2
8. Other	no

^a^Phrased as in Marshall’s criteria; ^b^modify Marshall’s criteria

Through the Nominal Group Technique we obtained a new set of classification.

criteria (Table 2).

**Table 2:** New set of PFAPA classification criteria and Modified Marshall’s classification criteria with number of patients that didn’t fulfill each criterion

New classification criteria	Patients excluded (N)	Modified Marshall’s classification criteria	Patients excluded (N)
1. Periodic Fever for at least 6 months: a. Daily fever of at least 38.5 °C (axillar) for 2 to 7 days b. At least 5 regularly recurring fever episodes with maximum of 2 months interval between them	20	Regularly recurring fevers with an early age onset (< 5 years of age)	7
2. Pharyngitis, cervical adenitis, oral aphthae: *at least one* in every episode and *at least 2 out of 3* in the majority of episodes.	21	2. Constitutional symptoms in absence of upper respiratory infection with at least one of the following clinical signs: • apthous stomatitis • cervical lymphadenitis • pharyngitis	0
3. Exclusion of other causes of recurrent fever (clinical or by laboratory depending on history)	0	3. Exclusion of cyclic neutropenia	0
4. Exclusion of infections, immunodeficiency and cyclic neutropenia	0		
5. Disease onset before the age of 6 years	1		
6. Full recovery between episodes	2	4. Completely asymptomatic interval between episodes	2
7. Normal linear growth	4	5. Normal growth and development	3

Different version proposed for some variables were ranked by participants to choose the most appropriated one (e.g. duration of attacks: > 2 days, > 3 days, < 5 days, 3–6 days, max 5 days, or max 6 days).

Periodicity of the febrile episodes as well as the maximum interval between episodes were specified in the new criteria, as well as the mandatory presence of at least one sign during each episode or even 2 in most episodes.

In the new criteria age at disease onset was modified in the new criteria at < 6 years instead of < 5 years, and more detailed description of other causes that clinician has to exclude were provided (other causes of recurrent fever, infection and other immunodeficiency than cyclic neutropenia). The new criteria specified that the pattern of growth have to be linear. After discussion, response to steroid was considered as supportive criteria and not included in the new criteria.

Both the newly proposed PFAPA criteria and the Marshall criteria were applied to a group of 80 PFAPA patients followed in Lausanne and Genoa in order to test their performance. The characteristics of patients included in the analysis are shown in Table 3. The proportion of boys and girls was 1:0.8. The median age at onset of PFAPA was 1.9 years old, the median age at diagnosis was 4 years.

**Table 3:** Characteristics of 80 PFAPA patients included in the analysis

Characteristic	
M/F	1.2
Age at onset (years)	Median 1.9 (IQR 0.8–3.1)
Age at diagnosis (years)	Median 4 (IQR 2.6–6)
Pharyngitis	88.7%
Adenitis	85.0%
Aphthous Stomatitis	71.2%
Headaches	30.0%
Digestive:	
• Nausea	6.25%
• Vomiting	20.0%
• Abdominal pain	48.7%
• Diarrhea	22.5%
• Hepatomegaly	1.2%
• Splenomegaly	7.5%
Arthralgia	33.7%
Arthritis	1.2%
General malaise	37.5%
Skin rash	21.2%
Conjunctivitis	8.7%
Myalgia	26.2%
Neurological symptoms (febrile seizure, one encephalitis)	5%
Acute scrotum	1.2%
Peritonitis	0%

All patients presented at least one of the 3 cardinal symptoms: aphthous stomatitis, pharyngitis, and cervical adenitis. Abdominal pain was the most frequently associated symptom, found in nearly 50% of the patients.

Only 41/80 patients (51%) fulfilled the new criteria. 31 patients were excluded because they didn’t meet one of the classification criteria, 7 because of 2 criteria, and 1 because of 3 criteria. Application of both criterion 1 and 2 from the new criteria to our cohort resulted in the exclusion of 36/39 (92%) patients.

Current modified Marshall criteria were met by 69/80 patients (86%). 7/80 didn’t fulfill the Marshall criteria because of the age at onset (after 5 years of age).

By comparing patients excluded by the two sets of criteria, we noted that 6/80 patients (7,5%) were excluded from both. Table 2 provides the number of patients not satisfying each criterion for both sets.

## Discussion

Thanks to a collaborative effort made by clinicians in the field of autoinflammatory diseases we developed a provisional new set of classification criteria for PFAPA disease based on experts’ opinion and we tested their performance in a cohort of PFAPA patients. The new criteria seem quite difficult to apply, since they need a precise history that can’t always be provided by parents. The characteristics of fever episodes provided in the new criteria are probably too restrictive and excluded nearly half of the patients. Conversely, the criterion “age at onset <6 years” seems better adapted and excluded only 1 patient [5]. There are not other substantial differences between new criteria and modified Marshall’s criteria. Globally, the new criteria were able to classify fewer patients as PFAPA in respect to the modified Marshall’s criteria if applied on our cohort of patients. By using these new criteria in clinical practice to diagnose PFAPA, a significant number of patients would not be diagnosed, and we consider them as not useful as diagnostic criteria. On the other hand, since they are very restrictive, these criteria probably would allow selecting a more homogeneous group of patients; therefore they may be suitable to select well-defined cohorts of patients for clinical research. In fact, Marshall criteria have been found to be poorly specific when tested in a population in which different monogenic periodic fevers are present [3]. This study presented some limitations and do not resolve the classification dilemma of PFAPA patients for different reasons. First of all, the elaboration of the new criteria was not the result of a statistical multivariate analysis of real patients with different confounding conditions (i.e. other autoinflammatory diseases, cyclic neutropenia or recurrent infections), but was made on the unique judgment of a panel of experts after the indications coming from a larger Delphi survey. The lack of a control group didn’t allow us to calculate specificity and sensitivity of the new criteria. Moreover, the evaluation of the new criteria was done retrospectively on a cohort of only 80 patients with the diagnosis of PFAPA coming from only two centers, for which the diagnosis was based on the judgments of each center, not on the basis of a consensus among different experts.

This was the first attempt to create a new set of classification criteria for PFAPA syndrome based on a consensus among experts. With its limitations, this study allowed us to identify difficulties hidden in a consensus-based process, underlying the need for a definition of gold standard PFAPA patients to develop validated classification criteria, and paving the way for a better-structured project that is ongoing.

## Conclusions

Our work led us to identify a new set of classification criteria for PFAPA syndrome that resulted to be too restrictive to be applied in daily clinical practice for the diagnosis of PFAPA. A new attempt, based on consensus among experts followed by evaluation and validation of the criteria on a cohort of gold standard patients is required, to define a set of classification criteria for PFAPA syndrome with a better performance in term of sensitivity and specificity.

## Abbreviation

PFAPA: Periodic fever aphthous stomatitis pharyngitis cervical adenitis
